# Long-term cultivation of grass–legume mixtures changed the assembly process of the microbial community and increased microbial community stability

**DOI:** 10.1093/ismeco/ycae157

**Published:** 2024-12-12

**Authors:** Huilin Yan, Xin Jin, Xueli Zhou, Songsong Gu, Xuexia Wu, Ping Li, Dejun Shi, Hanjiang Liu, Guangxin Lu, Ye Deng

**Affiliations:** Qinghai Provincial Key Laboratory of Adaptive Management on Alpine Grassland, Qinghai University, Xining, Qinghai, 810016, China; State Key Laboratory of Plateau Ecology and Agriculture, Qinghai University, Xining, Qinghai, 810016, China; College of Agriculture and Animal Husbandry, Qinghai University, Xining, Qinghai 810016, China; College of Agriculture and Animal Husbandry, Qinghai University, Xining, Qinghai 810016, China; Qinghai Province Grassland Improvement Experimental Station, Qinghai Provincial Forestry and Grassland Administration, Gonghe Qinghai 811800, China; Chinese Academy of Sciences Key Laboratory for Environmental Biotechnology, Research Center for Eco-Environmental Sciences, Chinese Academy of Sciences, Beijing 100085, China; State Key Laboratory of Plateau Ecology and Agriculture, Qinghai University, Xining, Qinghai, 810016, China; Academy of Agriculture and Forestry Science of Qinghai University, Qinghaidaxue, Xining 810016, Qinghai, China; Qinghai Province Grassland Improvement Experimental Station, Qinghai Provincial Forestry and Grassland Administration, Gonghe Qinghai 811800, China; Qinghai Province Grassland Improvement Experimental Station, Qinghai Provincial Forestry and Grassland Administration, Gonghe Qinghai 811800, China; College of Agriculture and Animal Husbandry, Qinghai University, Xining, Qinghai 810016, China; Chinese Academy of Sciences Key Laboratory for Environmental Biotechnology, Research Center for Eco-Environmental Sciences, Chinese Academy of Sciences, Beijing 100085, China

**Keywords:** grass–legume mixtures, microbial community, co-occurrence network, assembly processes

## Abstract

Grass–legume mixtures are a common cultivation system on the Qinghai–Tibet Plateau, where the interactions between rhizosphere microorganisms and crops under long-term cultivation are complex and dynamic. Investigating the dynamic changes in microbial community structure and ecological functions is essential. This study investigated the dynamic interactions of rhizosphere microbial communities of *Elymus nutans* Griseb. cv. Aba and *Medicago sativa* L. cv. Beilin in a grass–legume mixture at a 1:1 ratio >4 years on the Qinghai–Tibet Plateau. The research focused on their long-term effects on plant productivity, soil health, and microbial functions. The results revealed a decline in grass yield and soil properties in the fourth year (*P* < .05) and significant year-to-year differences in bacterial α-diversity (*P* < .05). Molecular ecological network analysis showed greater stability in the bacterial network of legumes in the first year, with reduced robustness by the fourth year. Additionally, the average niche widths of bacterial and fungal communities were narrower in the first year than in the fourth, indicating microbial adaptation to the evolving environmental conditions within the mixture system. The transition of bacterial community assembly processes from stochastic to deterministic suggests a shift toward more structured and predictable microbial interactions over time. In conclusion, the results highlight the intricate interplay between plant productivity, soil health, microbial community dynamics, and ecosystem stability under long-term planting of grass–legume mixtures. Our results provide new insights into biomass changes and microbial dynamics in this planting system.

## Introduction

The significant regionality and specificity of agricultural production on the Qinghai–Tibet Plateau, one of the world’s highest and most topographically complex plateau, is determined by the area’s high elevation and low temperature [1]. Grass–legume mixtures is a common planting system in this region [2]. Legume crops fix atmospheric nitrogen via symbiotic rhizobia, enhancing soil nutrients and reducing fertilizer reliance [3]. Crop diversification and interspecies interactions effectively minimize carbon emissions and nitrogen input in ecosystems. The interplanting of cereals and legumes significantly increases system productivity, microbial activity, and the activity of carbon (C) and (N) decomposing enzymes [4, 5]. Among the representative grassland types of the northern Qinghai–Tibet Plateau, *Elymus nutans* is a representative grass species [6]. After over a decade of cultivation and selection, we found that *E. nutans* Griseb. cv. Aba possessed the essential characteristics of high fat, high protein, high nitrogen-free extract, and low fiber contents. Additionally, it can withstand the adverse effects of the region’s extreme weather [7]. *Medicago sativa* L.cv (legume) is one of the three forage grasses commonly used to construct artificial grasslands on the Qinghai–Tibet Plateau [8]. Microbial diversity underpins nutrient cycling, plant health, and ecosystem function, offering insights into factors like soil fertility and productivity [9]. Rhizosphere microbial communities play a crucial role in plant growth, health, and yield [10]. Crop–microbe interactions evolve with cultivation time, soil conditions, and environmental factors [11]. Under long-term planting conditions, the structure and function of crop rhizosphere microbial communities often undergo dynamic changes [12]. Long-term planting alters nitrogen-fixing, phosphate-solubilizing, and plant growth promoting rhizobacteria abundances, impacting crop growth and health [13]. Moreover, microbial stability is vital for ecosystem functions, influencing grassland resilience to disturbances and climate change [14]. The interactions between rhizosphere microbes and forage under long-term planting conditions are complex and dynamic [11], and further attention to the dynamic changes in microbial community structure and their ecological functions under long-term grass–legume mixtures is essential. Therefore, studying the dynamic changes in microbial communities can provide a holistic perspective on how grasslands respond to management strategies, emphasizing the importance of sustainable practices for preserving ecosystem health and productivity. However, there are few studies on the dynamic changes within the microbial community under long-term planting of grass–legume mixtures in the Qinghai–Tibet Plateau.

Molecular ecological network analysis (MENA) serves as a powerful tool for investigating the potential interactions within microbial communities. It offers hypothetical insights into the complex interactions and dynamic shifts that potentially occur within microbial communities [15]. The long-term planting of grass–legume mixtures significantly affects the composition and function of rhizosphere microbial communities, influencing plant growth and health. In the short term, under grass–legume intercropping, significant changes occurred in the rhizosphere bacterial community, with legume plant-associated bacteria dominating. The bacterial networks are becoming more robust and more complex. The increased interactions, network stability, and complexity of the bacterial community align with the increase in aboveground biomass. The complexity and stability of the networks are significantly correlated with grass biomass, playing a pivotal role in influencing grass growth [16]. MENA constructs microbial ecological networks through high-throughput sequencing data, enabling the identification of key microbial species and their potential cooperative and competitive interactions [17]. The habitat niche breadth (*Bcom*) of a species refers to the range of resource utilization and the breadth of lifestyle it occupies within an ecosystem. *Bcom* reflects the species’ adaptability to available resources and its competitive ability in the environment. The *Bcom* of species plays a crucial role in the stability and diversity of ecosystems, which can increase a species’ adaptability and competitiveness in response to environmental changes, promote species coexistence, and maintain diversity. Therefore, studying niche width helps us understand the species’ interactions and competitive relationships. However, there is currently limited research on the niche width of bacterial and fungal communities under long-term planting of grass–legume mixtures.

This study utilized high-throughput 16S ribosomal ribonucleic acid (rRNA) and ITS gene sequencing and MENA to systematically investigate the effects of four consecutive years of grass–legume mixture planting on rhizosphere microbial communities and their interactions. We hypothesized that long-term planting of grass–legume mixtures (i) alters the rhizosphere microbial communities of grasses and legumes, (ii) changes the stability of rhizosphere microbial networks, (iii) modifies the assembly processes of rhizosphere microbes, increasing the overall community stability.

## Materials and methods

### Study sites and sampling

The experimental site was located at Qinghai Province Grassland Improvement Experimental Station, 99°35′ E, 37°0′ N, at an elevation of 3270 m. The annual average temperature was −0.7°C with a 20-day frost-free period. An average annual precipitation rate of 368.11 mm, a yearly evaporation rate of 1495.3 mm, and 58% relative humidity. The soil type was dark chestnut soil (Classification and codes for Chinese soil GBT17296-2009), rich in nutrients including total nitrogen (TN; 1404.7 mg/kg), ammonia nitrogen (4.58 mg/kg), nitrate nitrogen (NO_3_^−^-N; 42.17 mg/kg), and organic matter (SOM; 3.52%).

The grass used in this experiment was *E. nutans* Griseb.cv. Aba (Aba) from Sichuan Chuancao Ecological Grass Technology Development Co., Ltd., with a germination rate of >80% and a clearness of >90%, reaching the national secondary seed standard. The legume was *M. sativa* L*.*cv. Beilin 201 (Beilin 201) from Gansu Jiuquan Shengyuan Ecological Agriculture Company, with a germination rate of >80% and a clarity of >90%, reaching the national secondary seed standards. The experiment was sown around May 10, 2020, plot size was 3 m × 5 m, with an area of 15m^2^. Each plot was planted in 10 rows, the row spacing was 30 cm, and the sowing depth was 3 cm. Grass–legume mixture plots were sown with a grass, *E. nutans* Griseb. cv. Aba and treated with a legume *M. sativa* L.cv. Beilin 201 at a ratio of 1:1. Beilin201 was seeded at 45 g/15 m^2^, while Aba was seeded at 22.5 g/ m^2^, 6 replicates were set for each treatment. For all the following analyses, Beilin 201 (Legume) represents *M. sativa* L.cv. Beilin 201; Aba represents *E. nutans* Griseb.cv. Aba (Table 1, Supplementary Fig. S1). All plots were managed in a similar manner: manual weeding was adopted in the first year of planting, no pesticides were applied to any plots during the four years, no mowing was done in the four years of planting, Beilin 201 was mowed during the flowering period beginning in the second year, and the Aba was mowed after seed maturity.

**Table 1:** The treatments and number of replicates in this experiment.

Treatment number	Sowing method	Proportion (%)	Replicates
HD1	Aba# + Beilin 201	50	6
HD2	Aba# + Beilin 201	50	6
HD3	Aba# + Beilin 201	50	6
HD4	Aba# + Beilin 201	50	6
DH1	Beilin 201# + Aba	50	6
DH2	Beilin 201# + Aba	50	6
DH3	Beilin 201# + Aba	50	6
DH4	Beilin 201# + Aba	50	6

Note: Beilin 201 (Legume) represents *M. sativa* L.cv. Beilin 201; Aba represents *E. nutans* Griseb.cv. Aba; #: one of the grass–legume mixtures. 1: first year, other years are numbered in sequence.

Residues and impurities on the soil surface were meticulously cleared within the designated quadrats, where aboveground biomass measurements were obtained. Soil samples were collected from a depth range of 0 to 20 cm at each specific plot, carefully mixed, and placed into individual zip lock bags labeled with sample numbers. Multiple soil samples were randomly gathered from various locations within the plot, with five samples collected per plot (*n* = 5). These individual samples were combined to create a composite sample representing each plot. In total, 48 composite soil samples were obtained, HD = grass samples, DH = legume samples. A portion of each collected soil sample was utilized to analyze soil physical and chemical properties, while the remaining portion was stored at −80°C for subsequent Deoxyribonucleic acid (DNA) extraction.

### Soil physicochemical analyses

Soil parameters, such as TN, SOM, NO_3_^−^-N, available phosphorus (AP), and available potassium (AK) and ammonium nitrogen (NH_4_^+^-N), were assessed using established chemical analysis methods. The soil TN content was conducted using the Kjeldahl nitrogen determination method. The SOM content was determined using the potassium bichromate and oil bath heating method. NH_4_^+^-N content was quantified using the indophenol blue colorimetry technique, while NO_3_^−^-N content was measured using ultraviolet spectrophotometry. Enzyme hydrolysis was used to determine total phosphorus (TP) in the soil, while sodium hydroxide fusion was employed for total potassium measurement using flame photometry [16].

### Deoxyribonucleic acid extraction, amplification, sequencing, and sequence analysis

The total DNA of the soil microbial community was extracted using the soil DNA extraction kit (MOBIO Laboratories, Carlsbad, CA, USA). DNA concentration was determined using the NanoDrop-ND1000 spectrophotometer, and the quality of DNA samples was assessed through 2% agarose gel electrophoresis. Qualified DNA samples were then used for library construction. The total DNA extracted from soil samples served as a template for polymerase chain reaction amplification using specific primers with 10-bp barcode sequences. For the amplification of the 16S rDNA V4 region, the primers used were 515F (5′-GTGCCAGCMGCCGCGGTAA-3′) and 806R (5′-GGACTACHVGGGTWTCTAAT-3′). For the amplification of the ITS2 region, the primers used were 5.8F (5′-AACTTTYRRCAAYGGATCWCT-3′) and 4R (5′-AGCCTCCGCTTATTGATATGCTTAART-3′) [16]. The KAPA HyperPrep Kit was employed for DNA library construction, and sequencing was performed on the Illumina Miseq platform (Shanghai, LingEn Biotech).

### Statistical analysis

In this study, richness, Shannon index, and phylogenetic diversity index were employed to assess microbial community diversity in the context of grass–legume mixtures. To evaluate dissimilarities in microbial community structure, Bray–Curtis distance was utilized in principal coordinate analysis (PCoA). Furthermore, multiple response permutation program, one-way analysis of variance, and permutational multivariate analysis of variance were adopted as statistical methods to compare the community structure across the first to the fourth year of the grass–legume mixtures (https://dmap.denglab.org.cn) [18]. The one-sample Student’s *t*-test was also employed to assess the significance of observed networks compared to random networks.

Using the “VennDiagram” package in R (4.4.1), Venn diagrams were created to show the shared and unique operational taxonomic units (OTUs) between the first to the fourth years grass–legume mixtures microorganisms [19]. Statistical analysis of differentially abundant OTUs within habitats was performed using the “*edgeR*” package in R. The abundance of OTUs in different habitats over different years was depicted using ternary plots.

To reveal the relationship between microbial communities and crop rotation patterns, we utilized a publicly available data analysis pipeline (https://inap.denglab.org.cn) to construct molecular ecological networks (MENs) [20, 21]. Using the random matrix theory (RMT) method, we constructed separate MENs for bacteria and fungi. We performed Spearman correlation filtering on the RMT results of microbial communities, with thresholds for bacteria and fungi set at r ≥ 0.90. To assess the significance of each observed MEN, we generated 1000 randomly reconnected networks using the Maslov–Sneppen method [22] and evaluated the topological properties of each index.

Additionally, to demonstrate whether and how the length of cultivation affects network stability, we calculated network robustness and vulnerability. Robustness is defined as the proportion of species remaining in the network after the random removal of 50% of the nodes [15]. Vulnerability is measured by calculating the vulnerability of each node to determine its relative contribution to global efficiency.

To help reveal patterns of environmental selection and its effects on microbial communities, we also estimated the Levin niche width (B) index of bacterial members in each population using the “niches” and “width” functions in the “*spaa*” package in R [23]. The community-level B-value (*Bcom*) was calculated as the average B-value of all OTUs occurring within a community [24]. The normalized stochasticity ratio based on phylogenetic beta diversity (p*NST*) can quantitatively assess the relative importance of deterministic and stochastic processes in community assembly, with values ranging from 0 to 1, where p*NST* < 0.5 indicates that deterministic processes dominate, and p*NST* > 0.5 indicates that stochastic processes dominate. The average variability degree (AVD) index was calculated to evaluate the stability of microbial communities [25]. In general, lower AVD values indicate higher community stability. To identify the main drivers of the community aggregation process, the relationship between community aggregation and microbial community stability was evaluated using regression analysis of p*NST* and AVD values. Changes in community aggregation along the gradient of selected environmental variables (soil nutrients) were assessed using regression analysis by comparing p*NST* values with the Euclidean distance matrix of soil nutrient variables. Soil nutrients, such as TN, TP, NH_4_^+^-N, NO_3_^−^-N, AP, SOM, and AK, were standardized using Euclidean distance as the nutrient matrix.

## Results

### Changes in biomass and soil variables

Analysis of variance indicated that the fresh weight of forage in the fourth year of grass–legume mixed cropping was significantly reduced compared to the first year (*P* < .05; Table 2). The soil physical and chemical results showed that soil NH_4_^+^-H, NO_3_^−^-N, AP, and OM were significantly lower in the fourth year compared to the first year (*P* < .05).

**Table 2:** Comparison of forage biomass and soil physicochemical properties over four consecutive years.

Years		First	Second	Third	Fourth
Forage biomass	Grass	11.21 ± 0.68^*^	12.08 ± 0.69	5.50 ± 1.34	3.86 ± 2.56^*^
	Legume	7.69 ± 0.01	13.32 ± 1.11	14.90 ± 3.89	6.12 ± 1.17
TN (mg/kg)		2435.63 ± 208.17	2176.15 ± 140.00	2192.46 ± 358.27	2206.21 ± 181.00
TP (mg/kg)		683.42 ± 121.58	606.69 ± 51.54	583.69 ± 52.54	596.59 ± 67.56
NH_4_^+^-N (mg/kg)		53.40 ± 21.91^*^	87.76 ± 19.19	46.71 ± 18.37	32.76 ± 9.01^*^
NO_3_^−^-N (mg/kg)		7.82 ± 2.65^*^^*^^*^	3.22 ± 0.64	2.76 ± 0.30	2.81 ± 0.10^*^^*^^*^
AP (mg/kg)		16.44 ± 1.68^*^^*^^*^	18.82 ± 5.77	14.49 ± 4.55	11.88 ± 1.71^*^^*^^*^
OM (%)		6.21 ± 0.51^*^^*^^*^	5.24 ± 0.82	5.33 ± 0.65	5.26 ± 0.48^*^^*^^*^
AK (mg/kg)		172.83 ± 31.59	149.17 ± 22.34	155.50 ± 32.05	141.00 ± 32.63

^*^^*^^*^ *P* < .001; ^*^P < .05

### Changes in bacterial and fungal communities

**Figure 1:**
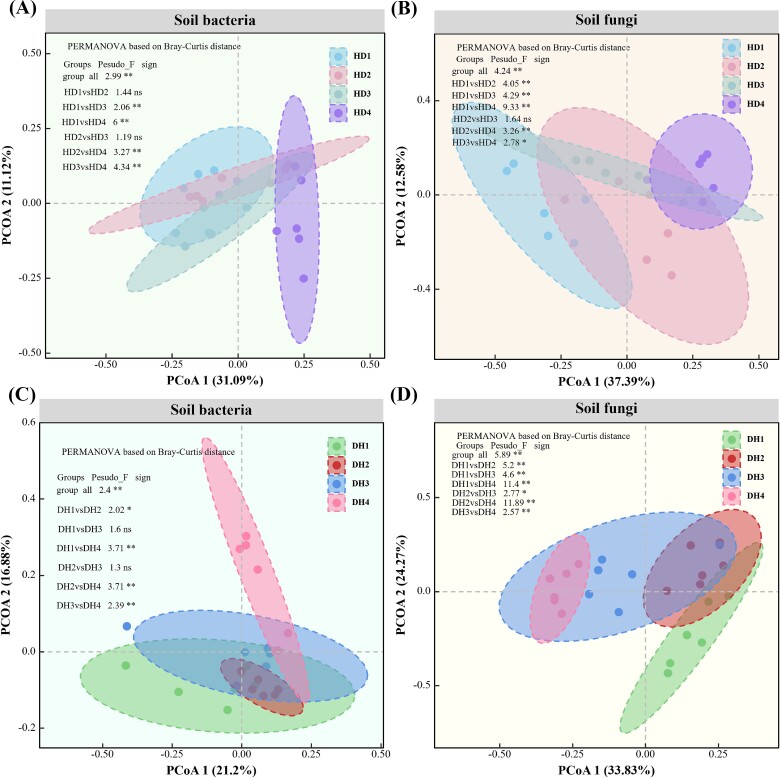
PCoA based on the Bray–Curtis distance matrix of bacterial and fungal communities over four consecutive years.

High-throughput sequencing of the 48 samples resulted in 4 187 945 bacterial and 4 157 171 fungal sequences, which could be clustered into 16 838 and 2550 OTUs at a 97% similarity level. The results showed significant differences in the bacterial and fungal communities between the first and fourth years for both legume (DH1 and DH4) and grass (HD1 and HD4; *P* < .05). PCoA based on the Bray–Curtis distance matrix indicated a clear separation of the bacterial and fungal communities between the first and fourth years (Fig. 1).

Significant differences in bacterial community richness and fungal community phylogenetic diversity were found between the first and fourth years of the grass communities (HD1 and HD4; *P* < .05). However, the α-diversity of the bacterial and fungal communities of legumes remained consistent over the same period (Supplementary Fig. S2). Analysis of rhizosphere β-diversity using Bray–Curtis distance-based testing indicated differences under varying lengths of cultivation (Supplementary Fig. S3). Overall, long-term planting of the grass–legume mixture influenced the microbial communities of both the legume and grass.

A total of 40 bacterial phyla and 554 genera were identified. Proteobacteria, Actinobacteriota, and Acidobacteriota were the dominant bacterial phyla, while Ascomycota and Mucoromycota were the dominant fungi phyla. Genera, such as *Pseudarthrobacter*, *Fusarium*, *Stagonosporopsis*, and *Leptosphaeria*, were prevalent (Supplementary Fig. S4). The number of unique bacterial and fungal OTUs decreased over the four years for both grass (Aba) and legume (Beilin 201). Grass had higher unique bacterial and fungal OTUs in the first year compared to the fourth year. Legumes also showed a decrease in unique OTUs over time (Supplementary Fig. S5).

### Impact of length of cultivation on soil microbial communities

MENs were built using 16S rRNA and ITS sequencing data to study microbial interactions in grass–legume mixtures over four years. The networks were constructed with consistent thresholds, 0.90 for bacteria and 0.90 for fungi. Topological analysis showed average path lengths ranging from 1.326 to 4.755, suggesting small-world network characteristics compared to random networks.

**Figure 2:**
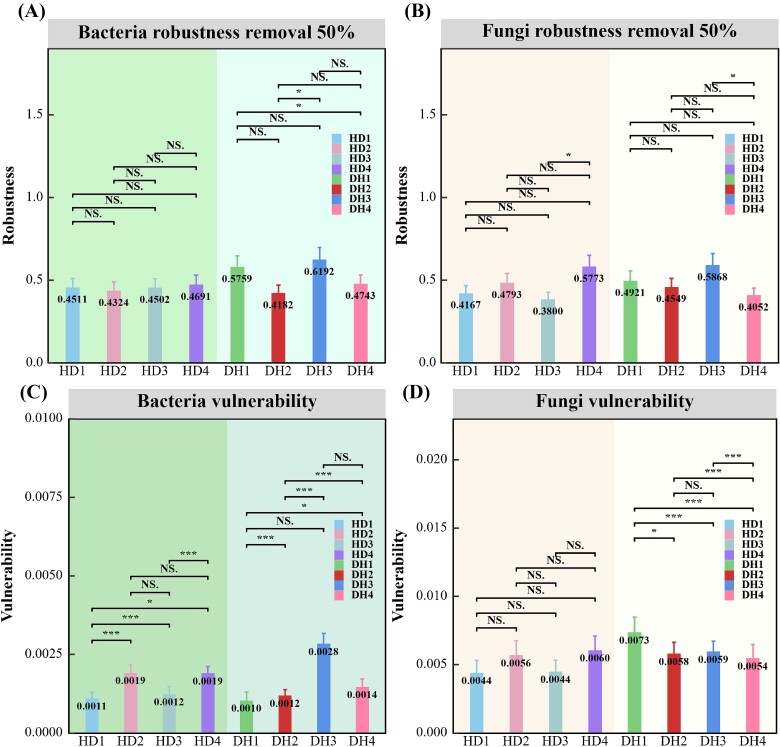
The complexity and stability of bacterial and fungal networks in grasses and legumes over four consecutive years. (A and B) Robustness of bacterial and fungal networks from grasses and legumes; (C and D) Vulnerability of bacterial and fungal networks in grasses and legumes. Asterisks indicate statistically significant differences between the two groups (^*^P < .05; ^*^^*^^*^P < .001; NS, not significant).

In the first year, both the bacterial and fungal communities of grass and legume had more nodes than the fourth year (Table 3). Network stability metrics, including robustness and vulnerability, were calculated for each network (Fig. 2). While no significant differences were observed in the robustness of the grass bacterial and fungal communities between the first (HD1) and fourth years (HD4), the legume networks showed a decrease in robustness from the first year (DH1: average 0.575 ± 0.010) to the fourth year (DH4: average 0.474 ± 0.002; Fig. 2A).

**Table 3:** Properties of the empirical and random MENs of the bacterial and fungal communities over four consecutive years of grass–legume mixtures. The randomized networks were generated by rewiring all nodes and links corresponding to the empirical networks 100 times.

Community	Years	Empirical networks							Random networks		
		Similarity threshold	Network size/nodes number	Connectivity/total links	Average degree (avgK)	Average clustering coefficient (avgCC)	Average Path distance (GD)	Modularity	Average clustering coefficient (avgCC)	Average path distance (GD)	Modularity
	HD1	0.997	647	808	2.484	0.781^a^	4.728^b^	0.978^c^	0.015	2.059	0.730
	HD2	0.977	668	778	2.620	0.772^a^	5.103^b^	0.985^c^	0.182	3.174	0.718
	HD3	0.985	612	738	2.543	0.821^a^	4.563^b^	0.986^c^	0.259	2.814	0.750
	HD4	0.997	578	698	2.365	0.887^a^	4.181^b^	0.974^c^	0.015	1.385	0.749
Bacteria	DH1	0.991	574	828	2.847	0.766^a^	4.341^b^	0.936^c^	0.026	0.696	0.647
	DH2	0.982	639	835	2.631	0.749^a^	0.949^b^	4.759^c^	0.049	1.581	0.671
	DH3	0.968	603	738	2.713	0.763^a^	0.948^b^	4.621^c^	0.154	1.242	0.629
	DH4	0.991	506	847	3.312	0.830^a^	0.932^b^	3.647^c^	0.034	0.433	0.579
	HD1	0.991	219	341	2.593	0.768^a^	0.918^b^	2.371^c^	0.026	2.804	0.594
	HD2	0.980	186	338	2.952	0.928^a^	0.905^b^	1.284^c^	0.038	0.915	0.561
	HD3	0.976	200	342	2.585	0.758^a^	0.911^b^	2.348^c^	0.053	1.542	0.591
	HD4	0.991	193	327	2.765	0.894^a^	0.906^b^	1.301^c^	0.034	2.139	0.547
Fungi	DH1	0.979	209	328	2.236	0.748^a^	0.926^b^	1.898^c^	0.028	2.565	0.586
	DH2	0.947	208	317	2.485	0.798^a^	0.925^b^	1.713^c^	0.034	2.542	0.565
	DH3	0.963	197	348	2.572	0.834^a^	0.902^b^	1.582^c^	0.051	2.515	0.551
	DH4	0.979	186	333	2.951	0.848^a^	0.832^b^	1.378^c^	0.063	2.410	0.529

a Significant difference in avgCC between empirical and randomized networks based on Student’s *t*-test.

b Significant difference in GD between empirical and randomized networks based on Student’s *t*-test.

c Significant difference in M between empirical and randomized networks based on Student’s *t*-test.

### Grass–legume mixtures assembly process analysis

**Figure 3:**
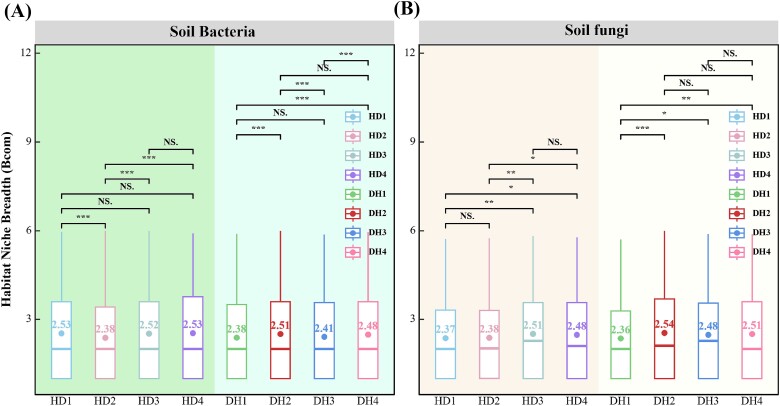
Habitat niche breadth (Bcom) of bacterial and fungal communities from grasses and legumes over four consecutive years of grass–legume mixture cultivation (^*^P < .05; ^*^^*^P < .01; ^*^^*^^*^P < .001; NS, not significant).

**Figure 4:**
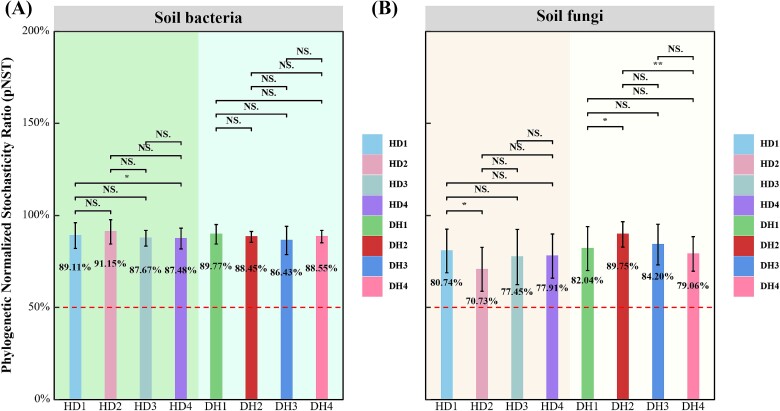
Comparison of p*NST* ratio of bacterial and fungal communities in grasses and legumes under grass–legume mixture cultivation over four consecutive years (^*^P < .05; ^*^^*^P < .01; NS, not significant).

The habitat niche breadth at the community level (Bcom) reveals the contribution of environmental selection to microbial community assembly. The Bcom of the grass fungal community was lowest in the first year. Similarly, the average habitat niche Bcom values of both the legume bacterial and fungal communities were also lowest in the first year (Fig. 3). The p*NST* values indicated that the assembly processes of the bacterial and fungal communities in both the first and fourth years were more influenced by stochastic processes. The stochastic processes for grass significantly decreased from the first year (HD1, 89.11%) to the fourth year (HD4, 87.48%; Fig. 4A).

**Figure 5:**
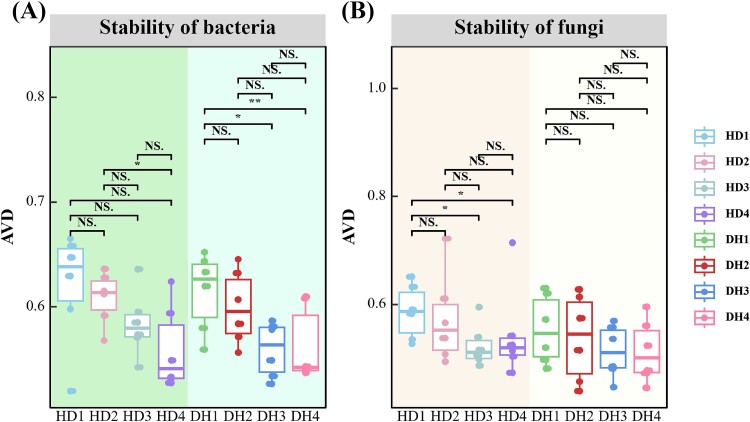
Analysis of microbial community stability over four consecutive years. (A) Bacterial communities; (B) fungal communities (^*^P < .05; ^*^^*^P < .01; NS, not significant).

**Figure 6:**
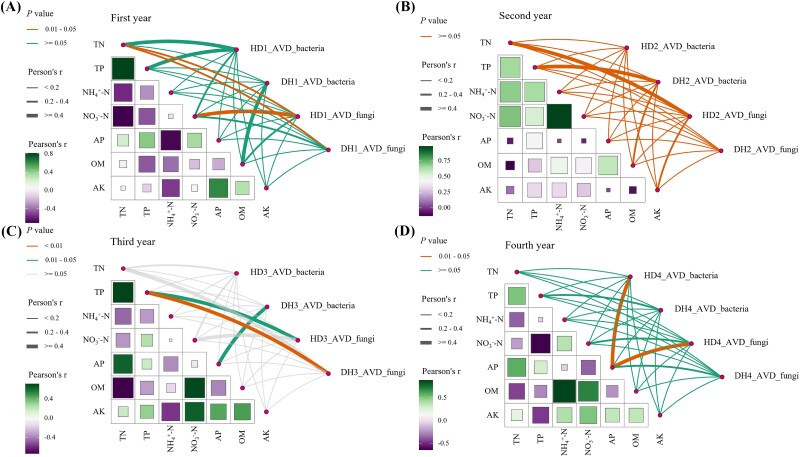
The relationship between microbial community stability and soil physicochemical properties. (A) First year; (B) second year; (C) third year; (D) fourth year.

Results indicated a significantly lower AVD in the legume bacterial community in the fourth year compared to the first year. Likewise, the AVD of the fungal community in grass was considerably lower in the fourth year compared to the first year (Fig. 5A and B). Additionally, we performed the Mantel test to examine the relationships between environmental factors and the AVD of the bacterial and fungal communities. The results indicated that in the first year, the AVD of the fungal community in the grass (HD1) was significantly correlated with NO_3_^−^-N and TN. In the fourth year, the AVDs of the bacterial and fungal communities in the grass (HD4) were significantly correlated with AP (Fig. 6A and D).

### Factors shaping microbial communities in mixtures

**Figure 7:**
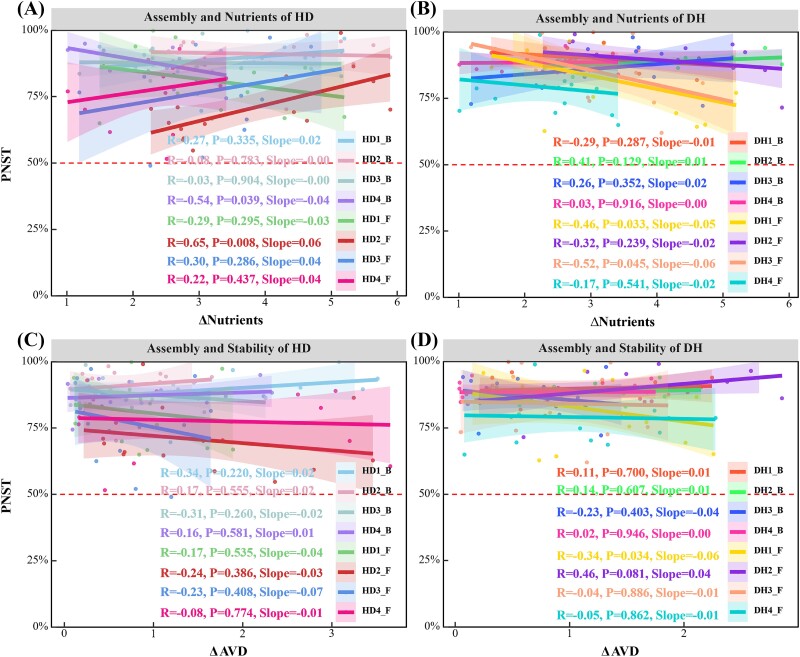
Linear regression relationship between microbial community p*NST* and AVD differences. Shaded areas represent 95% confidence interval. DH_B, where B stands for bacteria, and DH_F, where F stands for fungi. 1: first year, other years are numbered in sequence.

The linear fitting model demonstrated the relationships between p*NST*, soil nutrients, and community stability (AVD), inferring the relative influence of deterministic and stochastic assembly processes along environmental gradients (Fig. 7). In the assembly of the legume fungal community (DH_F), the continuous decrease in p*NST* indicates that as soil nutrient differences increase, community assembly shifts from being more stochastic to more deterministic (Fig. 7B). Correlation analysis between p*NST* and AVD revealed that for the legume fungal community (DH_F) under grass–legume mixtures, in the first year, as community stochasticity (*NST*) decreased, stability also reduced (Fig. 7D).

## Discussion

### Grass–legume mixtures reduced forage biomass and soil properties

Forage biomass in the fourth year of grass–legume mixture cultivation was significantly decreased compared to the first year, indicating a declining trend with increasing years of cultivation. This aligns with prior research, implying that prolonged plant intercropping could redistribute ecosystem resources and deplete soil nutrients [26]. The reduction in forage biomass may stem from declining soil nutrients. Grass–legume mixtures initially boost soil nitrogen levels through fixation but can deplete nitrogen (such as NH4^+^-N and NO3^−^-N) over time due to high plant demand [27]. Decreases in AP and OM further limit plant growth and yield [28]. Diminished SOM not only hampers nutrient supply but also harms soil structure and water retention. Soil health and fertility are linked to SOM decline, affecting productivity [29]. Plant competition in mixed cropping affects yield, with dominant species depleting resources, ultimately reducing overall productivity [30].

### Grass–legume mixtures alter the diversity and structure of microbial communities

Our study examined bacterial and fungal community diversity in grasses and legumes over four years to assess the effect of length of cultivation on rhizosphere microbial populations. Significant differences in bacterial richness and fungal phylogenetic diversity were observed between different planting periods, with those in grasses being particularly notable. Legumes displayed consistent α-diversity over the years, likely due to their nitrogen-fixing ability to support microbial stability [31, 32]. In contrast, grasses exhibited varied microbial community richness and diversity, indicating their susceptibility to environmental changes. Differential tests highlighted distinct β-diversity in the microbial populations across the length of cultivation, emphasizing structural shifts beyond changes in abundance. The separation of microbial communities between the years underscored temporal evolution influenced by plant growth, environmental factors, and climatic conditions [33]. Unique bacterial and fungal OTUs decreased over time for both grass and legume, suggesting a decline in specific rhizosphere species possibly due to environmental stress and plant-microbe interactions [32, 34].

### Grass–legume mixtures impact network stability and complexity of different forages

Through MENA, this study further reveals the impact of planting duration on the internal structure and stability of rhizosphere microbial communities in herbaceous grasses and legumes plants. Significant differences were observed in the number of nodes, network complexity, robustness, and vulnerability of the bacterial and fungal communities across various plants and length of cultivation. Initially, the microbial networks exhibited higher complexity in the first year, indicating closer microbial connections, possibly due to dynamic plant-microbe symbiotic relationships [35]. Over time, the microbial communities likely decreased in complexity due to competition and environmental pressures [34, 36].

In terms of network stability, grass showed consistent robustness over time, possibly attributed to stable root exudates supporting the microbial networks [37, 38]. In contrast, legume displayed increased stability in microbial networks in the fourth year, despite reduced complexity, indicating tighter and more robust structures less sensitive to external disturbances [39].

### Grass–legume mixtures change microbial community assembly, broadening niche width, and enhancing stability

The Bcom of bacterial and fungal communities in grass and legume differed significantly between the first and fourth years. The initially narrower niches in the first year indicated lower adaptability, potentially due to specific environmental constraints [40]. By the fourth year, communities exhibited broader niches, suggesting increased adaptability and survival under various conditions post-adaptation. This expansion in niche width over time signifies enhanced adaptability and survival strategies, likely stemming from niche differentiation and adaptive evolution within the microbial communities [41]. New ecological niches and resource utilization strategies may make the entire microbial community more resilient [42, 43]. p*NST* values indicated that the assembly process of the bacterial and fungal communities is more affected by random processes. This means that random dispersal, extinction, and colonization events played a significant role in community assembly at these time points [44].

We also found that the AVD of the legume bacterial community was significantly lower in the fourth year than in the first year. This suggests that by the fourth year, the species composition of the legume bacterial community had become more stable, possibly due to prolonged environmental selection and interspecies competition, leading to dominance by more adaptable species [45–47]. Similarly, the AVD of the grass fungal community was significantly lower in the fourth year than in the first year, indicating that species composition within this community had also become more stable over time [48].

In the initial year, the AVD of the grass bacterial community was significantly correlated with NO_3_^−^−N and TN, highlighting the impact of nitrogen availability on community stability early on. Nitrogen plays a crucial role in shaping microbial communities due to its influence on plant-microbe interactions [49]. By the fourth year, the AVD of the grass bacterial community was correlated with TN, whereas the fungal community was correlated with AP, indicating a shift in nutrient influences over time. Long-term succession likely prompts communities to adapt their responses to environmental changes, potentially leading to increased phosphorus demand in fungal communities [50].

### Grass–legume mixtures modify microbial assembly, broaden niches, and enhance stability

The linear fitting model revealed the relationships among the standard deviation of p*NST*, soil nutrients, and AVD, illustrating a continuous decrease in p*NST* with increasing soil nutrient differentiation. This decline suggests a shift in community assembly from more random to more deterministic processes. Under the grass–legume mixture, the stability of the legume fungal community was negatively correlated with its randomness, indicating that reduced randomness corresponds to decreased community stability. The interpretation of these results involves multiple theories and empirical studies in environmental and community ecology. Firstly, soil nutrients directly influence the structure and function of microbial communities [51]. Under high nutrient conditions, intense resource competition may favor dominant species capable of efficiently utilizing limited resources, leading to more deterministic community assembly. Conversely, under low nutrient conditions, lower resource utilization efficiency may increase the influence of random events on community structure, resulting in higher randomness in assembly [52, 53]. Secondly, community stability is closely related to the dynamic equilibrium of community structure [54]. In the rhizosphere of legume plants, fungal communities play a crucial role in supporting plant health and growth [10]. High randomness in community assembly may lead to instability in community function, causing fluctuating stability over time. In contrast, more deterministic assembly processes with fewer random factors can maintain relative stability in community function, thereby benefiting plant health [55–57].

In summary, with the increase in the length of grass–legume mixture cultivation, significant changes were observed in soil properties and microbial community structures. The stability of microbial network communities decreased, while the niche width and overall stability of bacterial and fungal communities increased. The assembly process shifted from stochastic to deterministic. This study provides scientific evidence for optimizing rhizosphere microbial communities in the planting and management of artificial grasslands in alpine regions, mainly through the long-term use of grass–legume mixtures. Understanding the dynamic processes of microbial community changes in grassland ecosystems and how environmental factors drive these changes can help reveal the fundamental ecological principles of grassland ecosystems. These findings not only aid in uncovering the basic ecological laws of grassland ecosystems but also provide crucial scientific evidence for ecosystem management and conservation.

## Supplementary Material

Figure_S1_ycae157

Figure_S2_ycae157

Figure_S3_ycae157

Figure_S4_ycae157

suppementary-end-jv_ycae157

## Data Availability

The original contributions presented in the study are publicly available. Data has been deposited in the China National Microbiology Data Center (NMDC) with the accession number NMDC10019010. URL is https://nmdc.cn/resource/en/genomics/sample/detail/ NMDC10019010. Data is deposited in the NMDC with the accession number NMDC40060475 (https://nmdc.cn/resource/genomics/metagenome/detail/NMDC40060475).
