# Supplementary figures and images for: Long-term cultivation of grass–legume mixtures changed the assembly process of the microbial community and increased microbial community stability
Source: ISME Commun. 2024 Dec 12;5(1):ycae157. doi: 10.1093/ismeco/ycae157 (PMC11879099; doi:10.1093/ismeco/ycae157)

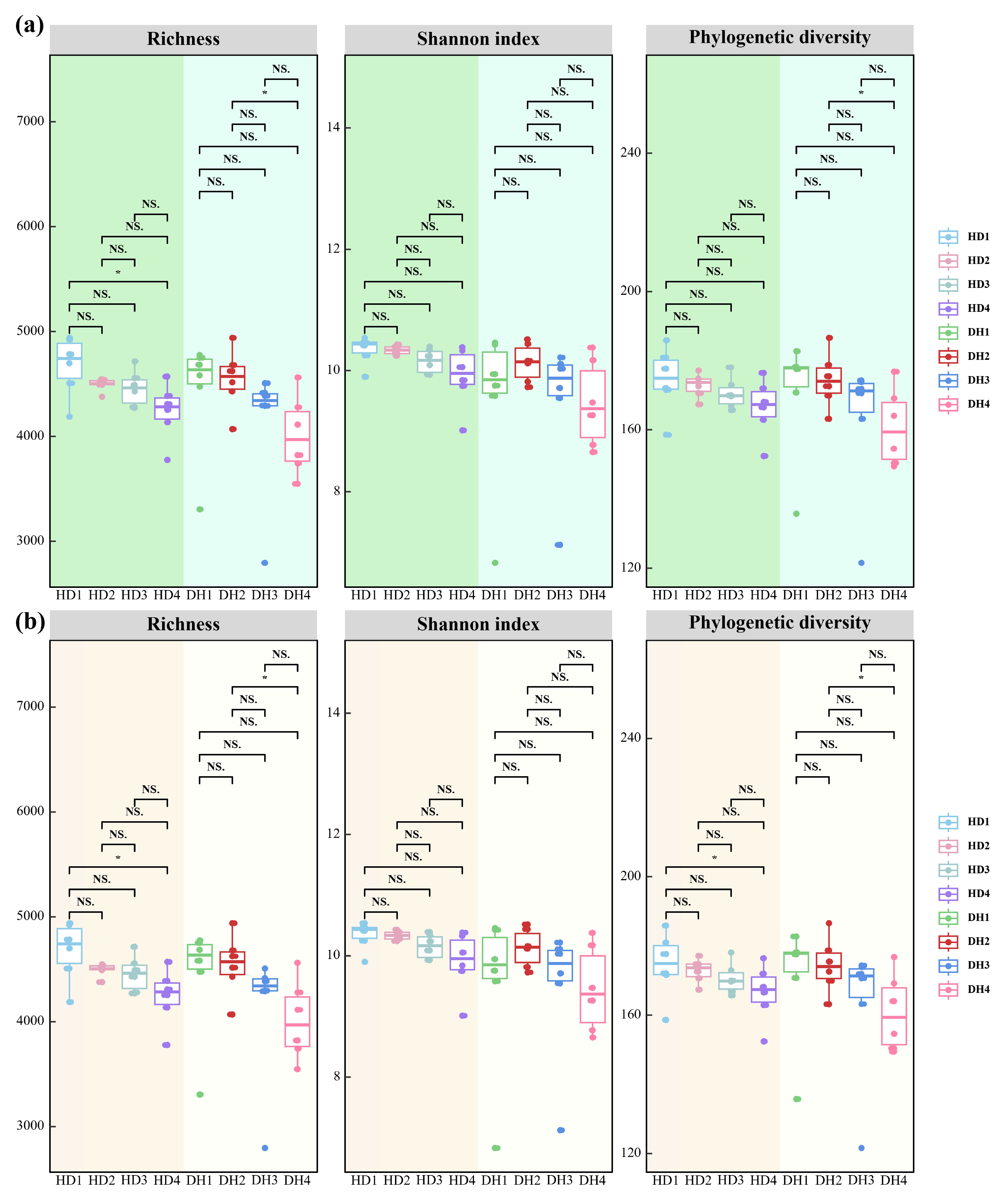

Supplement: Figure_S1_ycae157 [file figure_s1_ycae157.jpeg]

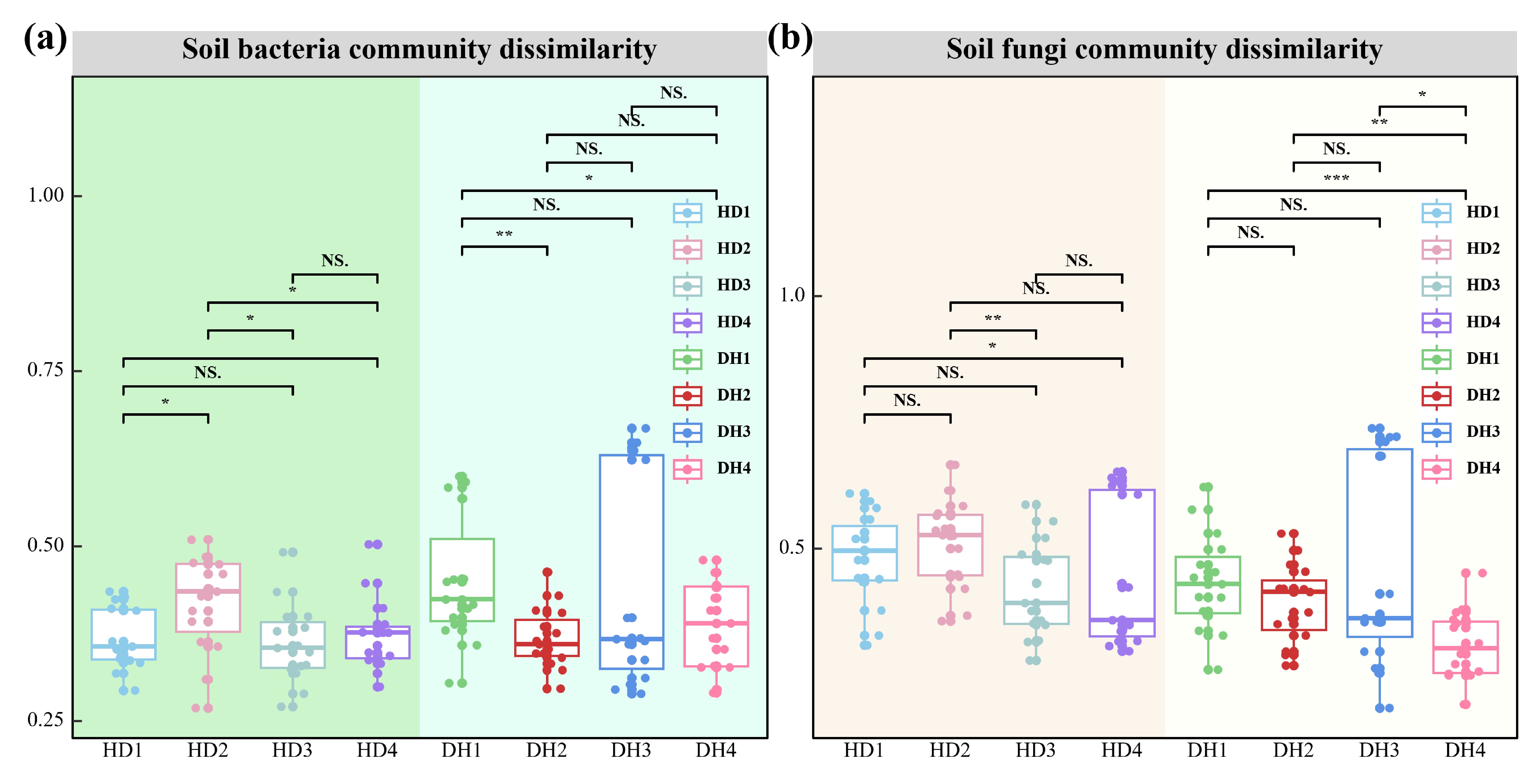

Supplement: Figure_S2_ycae157 [file figure_s2_ycae157.jpeg]

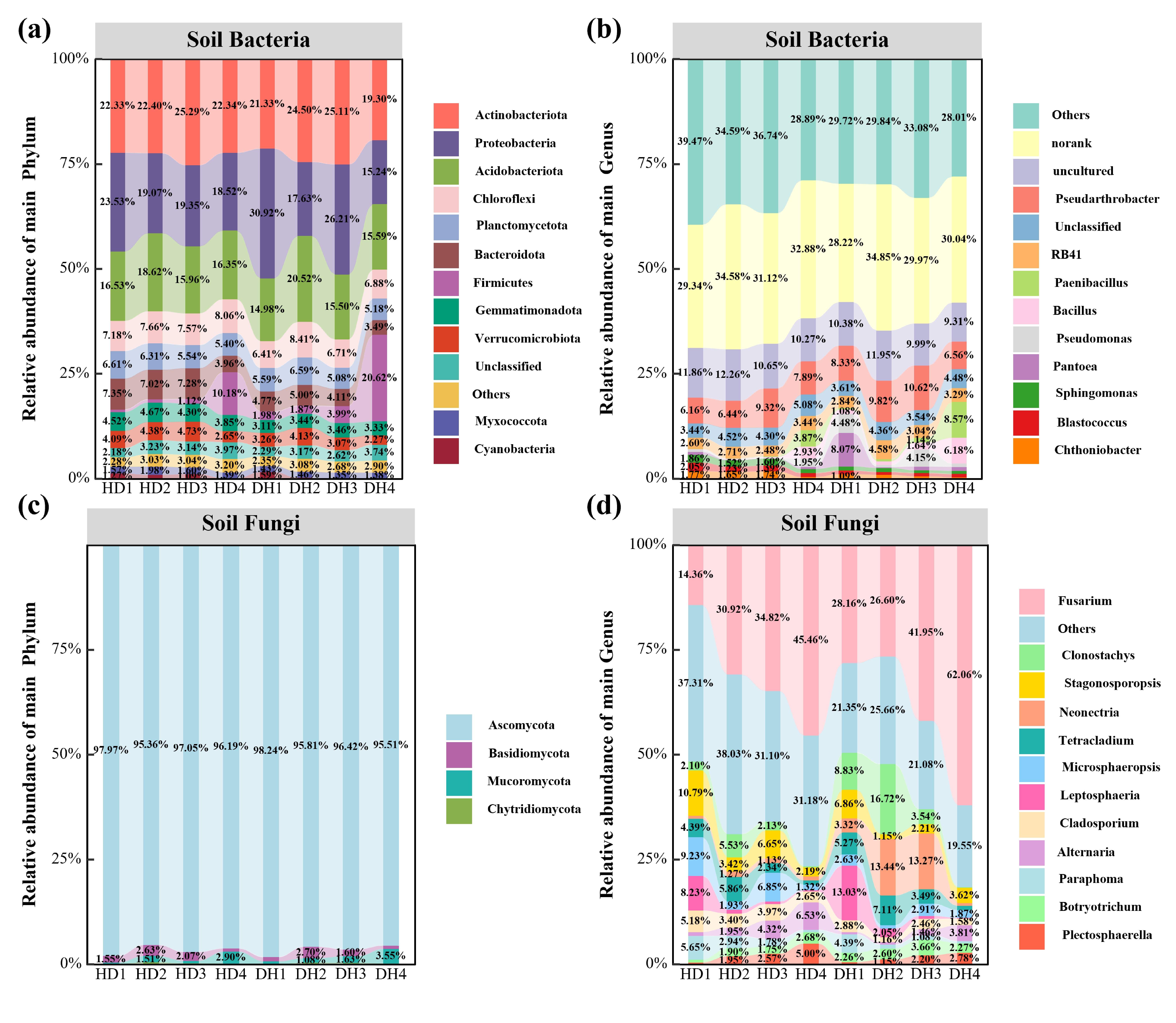

Supplement: Figure_S3_ycae157 [file figure_s3_ycae157.jpeg]

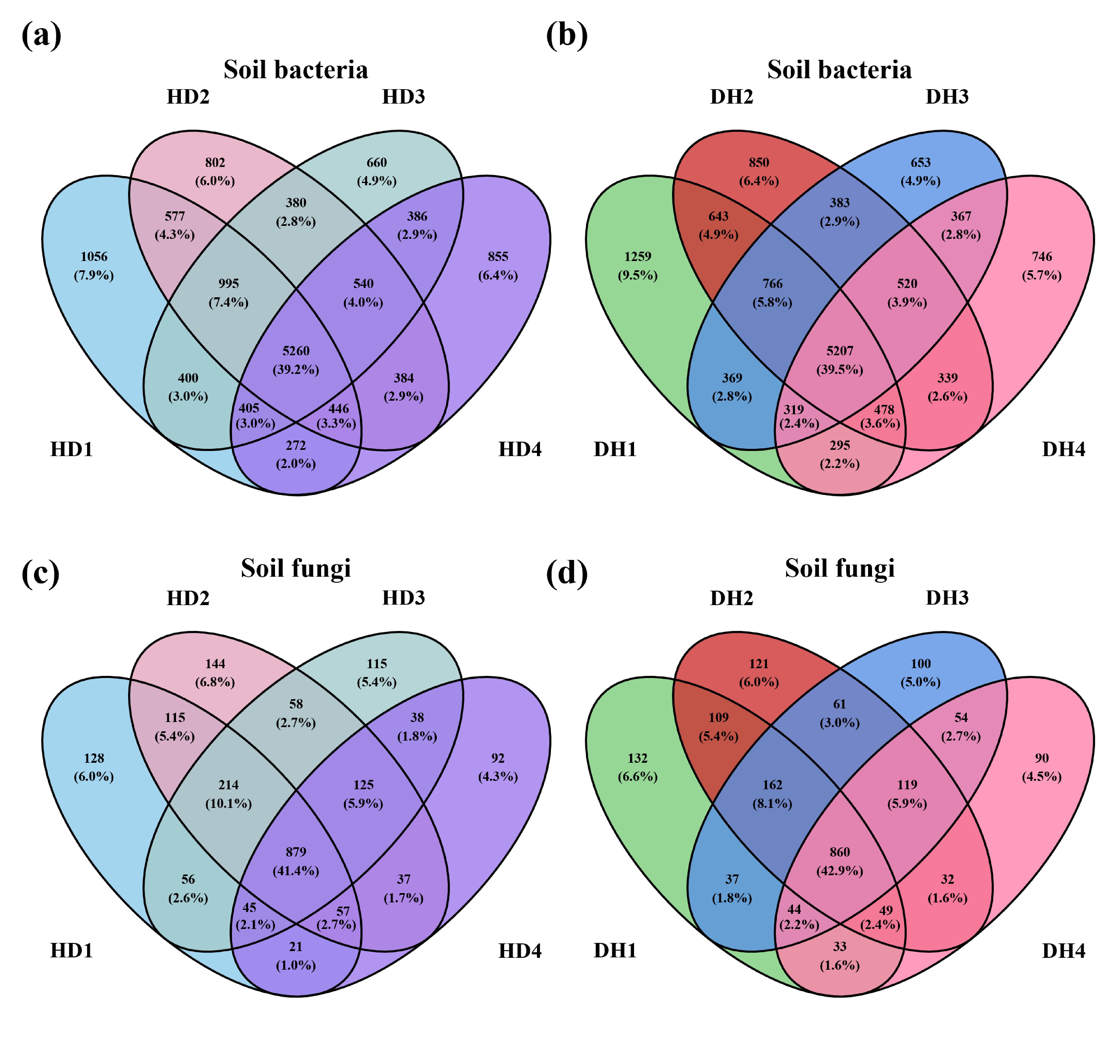

Supplement: Figure_S4_ycae157 [file figure_s4_ycae157.jpeg]
