# Supplementary material for: Long-term cultivation of grass–legume mixtures changed the assembly process of the microbial community and increased microbial community stability
Source: ISME Commun. 2024 Dec 12;5(1):ycae157. doi: 10.1093/ismeco/ycae157 (PMC11879099; doi:10.1093/ismeco/ycae157)

Figure S1 Aerial view of experimental design.


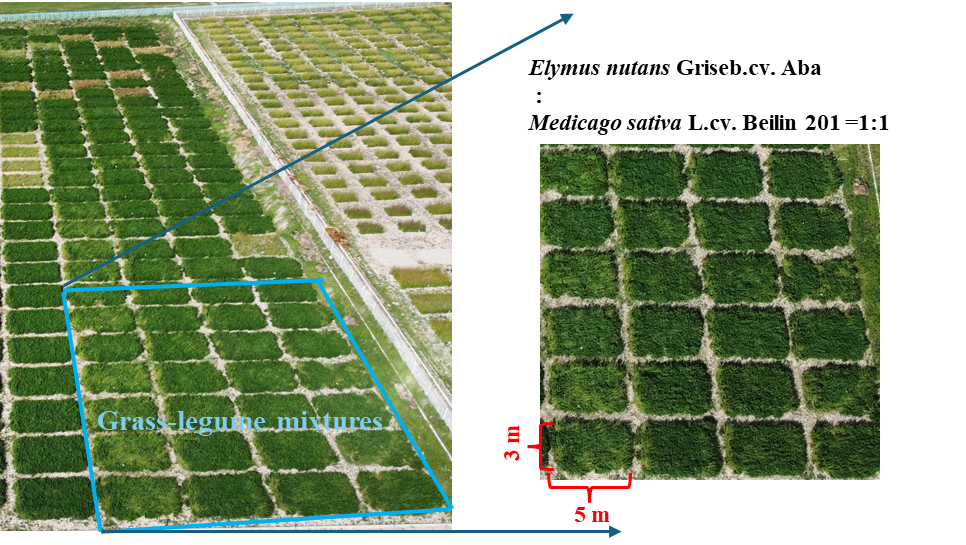


Figure S2 Comparison of α-diversity of bacterial and fungal communities in grasses and legumes under grass-legume mixtures over four consecutive years. (* P < 0.05, NS, not significant)


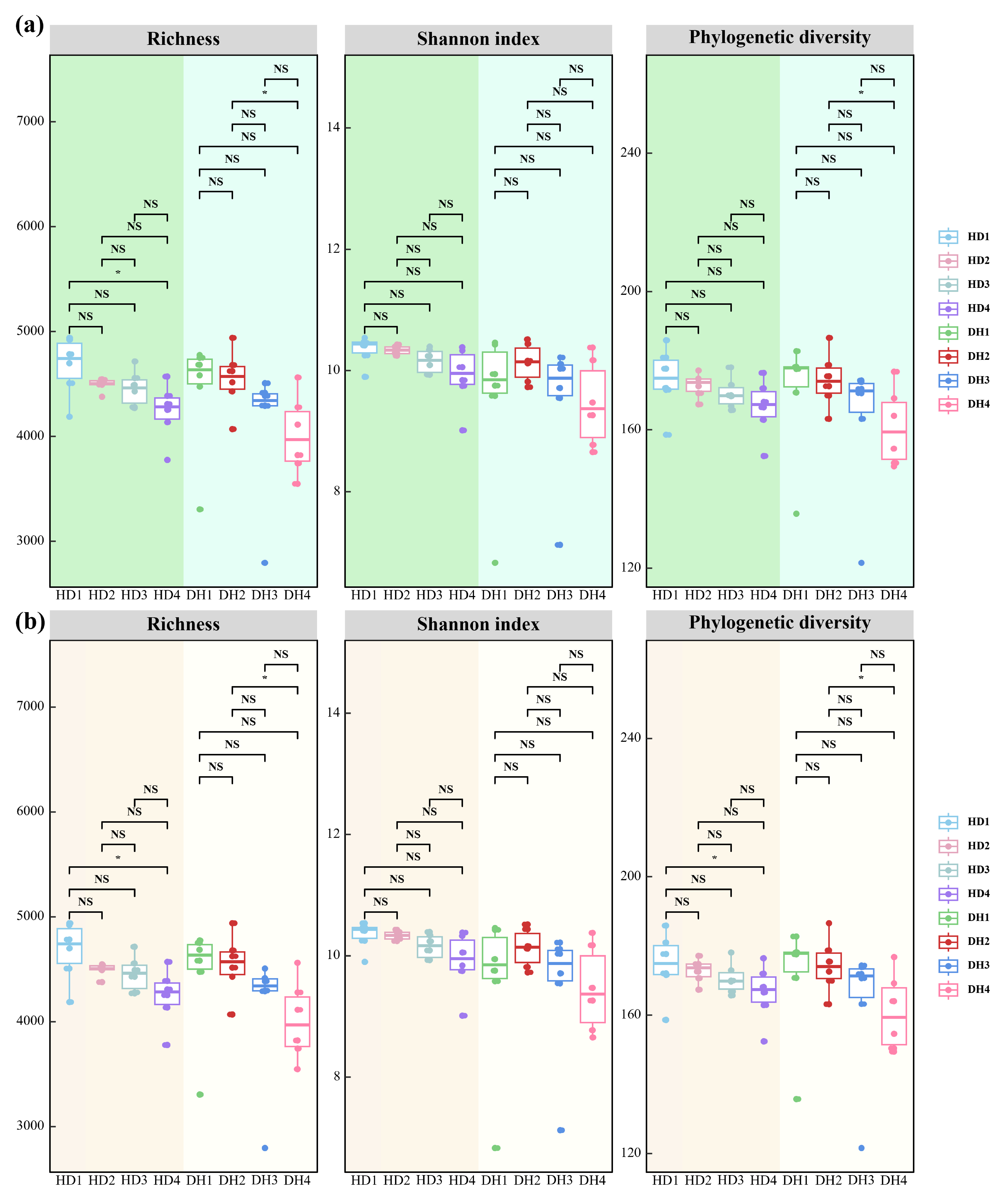


Figure S3 Dissimilarity of bacterial and fungal communities in grasses and legumes under grass-legume mixtures over four consecutive years (* P < 0.05, ** P < 0.01,***P < 0.001, NS, not significant)


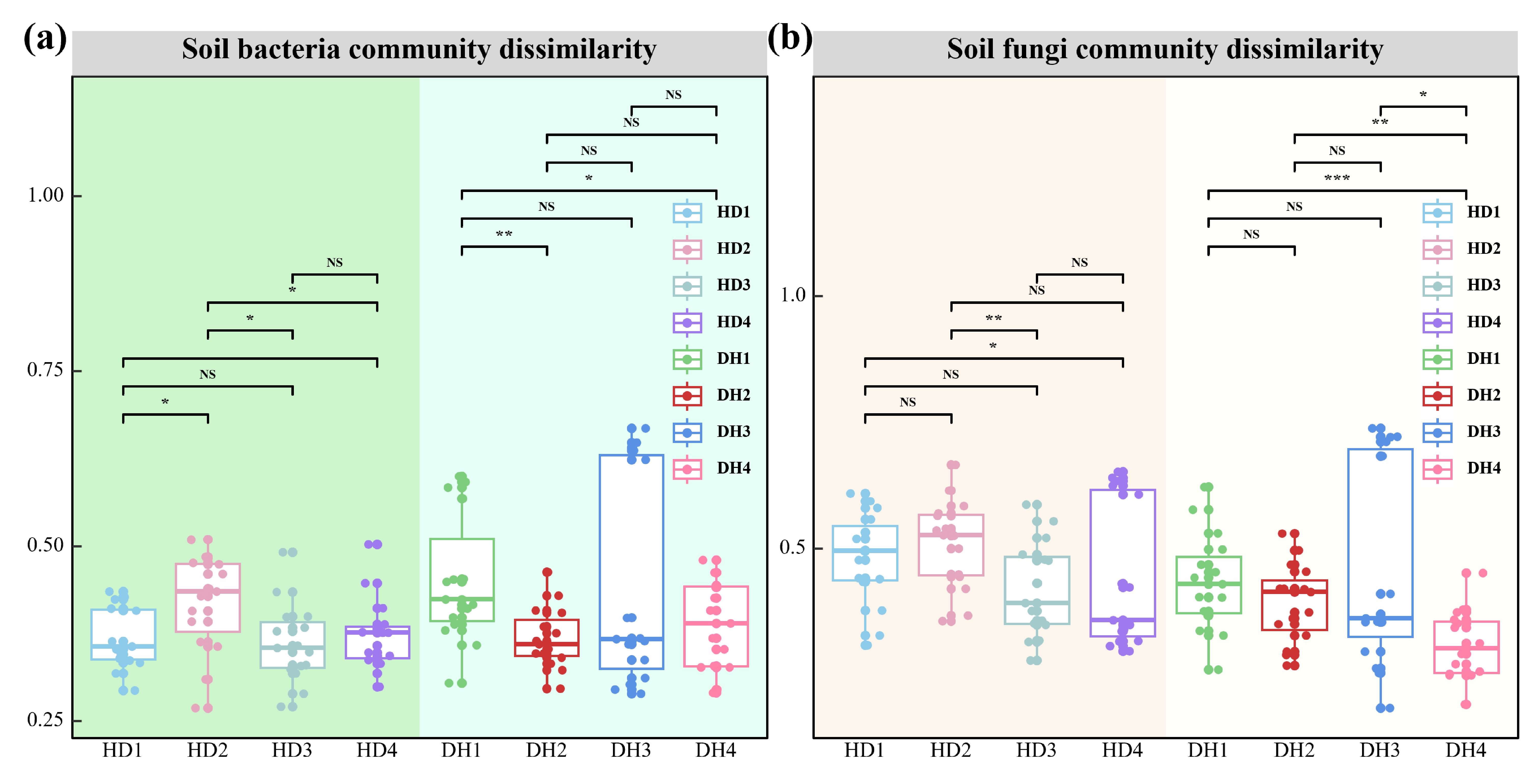


Figure S4 Species composition of bacterial and fungal communities in grasses and legumes under grass-legume mixtures over four consecutive years


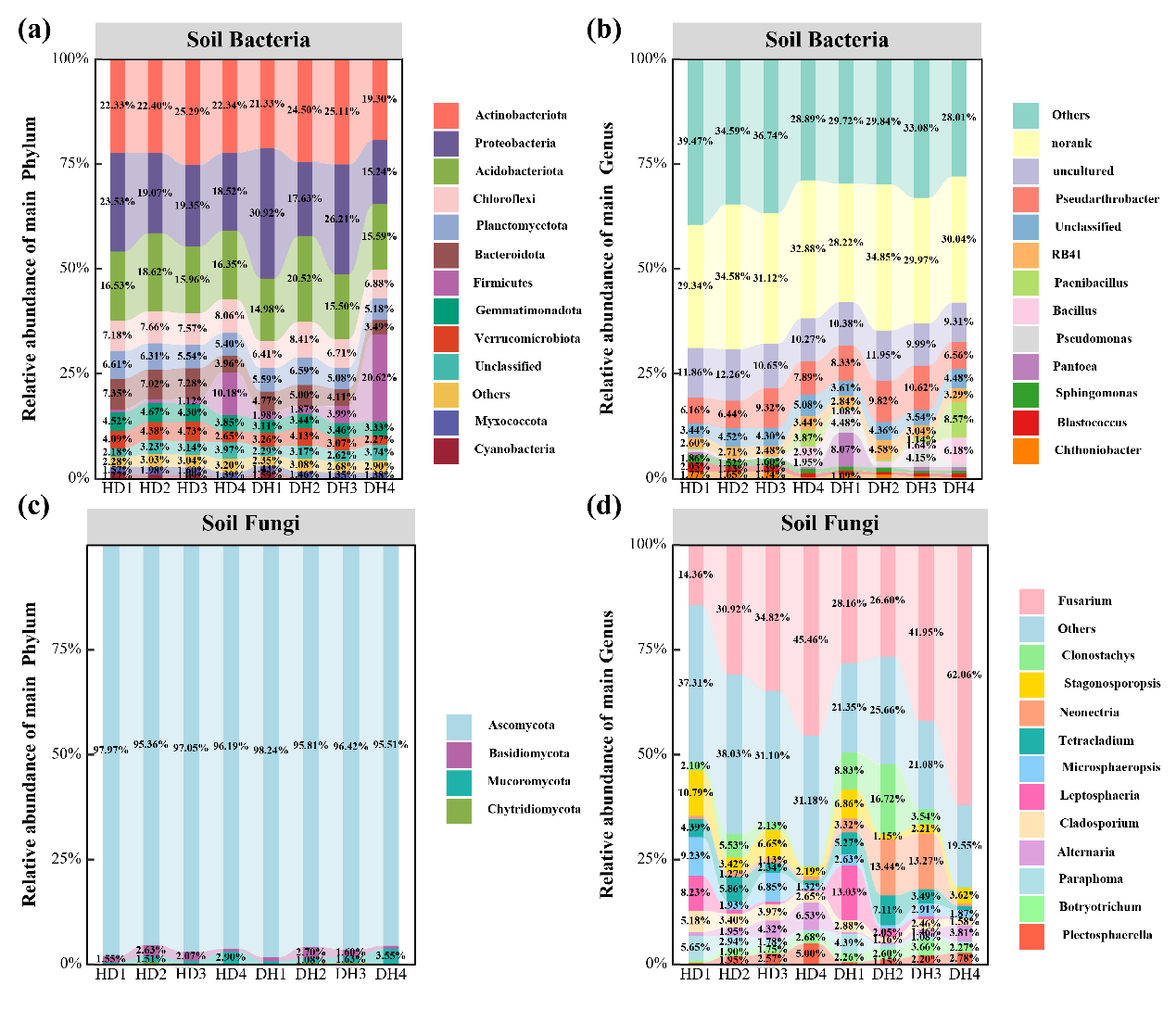


Figure S5 Venn diagram of unique and shared OTUs in bacterial and fungal communities of grasses and legumes under grass-legume mixtures over four consecutive years


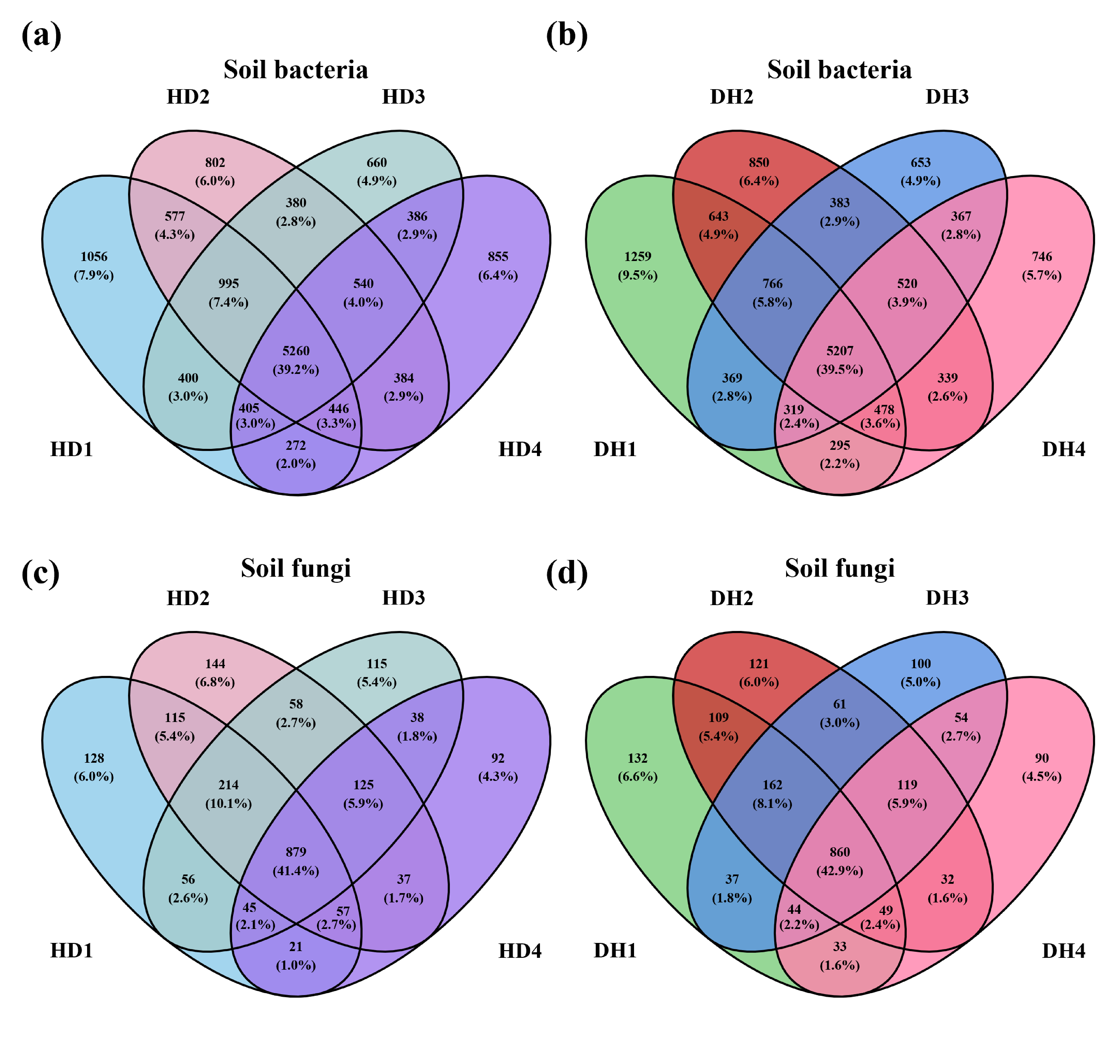

Supplement: suppementary-end-jv_ycae157 [file suppementary-end-jv_ycae157.docx]
